# Antituberculous Serum

**Published:** 1899-04-22

**Authors:** 


### ANTITUBERCULOUS SERUM.

The very interesting paper by Dr. Theodore Williams and Dr. Horrocks on the Use of Antituberculous Serum, which was read at the last meeting of the Royal Medical and Chirurgical Society, was one to raise a certain amount of hope in regard to the treatment of such early cases of tuberculosis as cannot from their position in life obtain the best treatment, namely, that by pure mountain air. We may pass over the first series of cases as not showing much, but those in the second series were certainly good. All four patients gained weight, the two excavation cases most, one 12½ lbs. and one a stone; the tuberculisation cases 5½ and 6lbs. respectively. All increased in strength and vigour and were able to return to their work, except one in whose case a lighter occupation was substituted for a comparatively trying one. Cough and expectoration diminished in all, and in one case altogether ceased. Physical signs showed limitation of the area of the disease in the consolidation cases, and in the two excavation cases quiescence of the cavity in one and complete contraction in the other. That in an early case the use of an antituberculous serum should tend to produce, or to hasten the production of immunity, is only what one is led to expect by the behaviour of the antitoxins of other diseases; but the effect of the injections on the excavation cases is extremely interesting, for it would seem to show that, if one can but place some check upon the activity of the tuberculous process, the secondary infections, by which ulceration and excavation are so largely caused, lose much of their power for evil.

